# Repeated aminoglycoside exposure activates interferon-stimulated genes in human renal proximal tubular models

**DOI:** 10.1007/s00204-026-04494-w

**Published:** 2026-07-14

**Authors:** Vidya Chandrasekaran, Tamara Meijer, Martin O. Leonard, Anja Wilmes, Paul Jennings

**Affiliations:** 1 Department of Chemistry and Pharmaceutical Sciences, Vrije Universiteit Amsterdam, Amsterdam Institute of Molecular and Life Sciences (AIMMS), De Boelelaan 1108, 1081 HZ Amsterdam, The Netherlands; 2 Toxicology Department, Radiation, Chemical, Climate and Environmental Hazards (RCCE) Directorate, Health Security Agency, Harwell Campus, London, OX11 0RQ UK; 3 Department of Chemistry and Pharmaceutical Sciences, Amsterdam Institute for Molecules, Medicines and Systems, Vrije Universiteit Amsterdam, De Boelelaan 1108, 1081 HZ Amsterdam, The Netherlands

**Keywords:** Aminoglycosides, proximal tubule, iPSC, megalin, interferon

## Abstract

Aminoglycosides remain clinically important antibiotics, but their use is limited by nephrotoxicity. Although proximal tubular accumulation mediated by megalin is well established, the downstream cellular response is still incompletely understood. Here, we compared repeated-dose transcriptomic responses to gentamicin and tobramycin in a panel of human proximal tubular models comprising primary proximal tubular epithelial cells (PTEC; three donors), human iPSC-derived proximal tubular-like cells (PTL; three donors), RPTEC/TERT1 cells, and HK-2 cells. Cells were exposed daily for seven days to 12, 250, or 450 µg/mL gentamicin or tobramycin, and gene expression was assessed using the TempO-Seq EU-ToxRisk v2.1 panel followed by DESeq2 analysis. Primary PTEC and PTL showed the clearest and most coherent response, characterised by strong induction of interferon-stimulated genes including IFI27, IFI6, IFIT1-3, ISG15, MX1, OAS1, UBE2L6, USP18, IFI44L, HERC6, and RSAD2. Targeted regulator analysis showed strong enrichment of overlapping ISGF3, IRF7, and IRF1 target networks, consistent with a dominant interferon-stimulated gene program, together with a smaller NF-κB-associated inflammatory branch. In contrast, RPTEC/TERT1 and HK-2 cells showed weaker and less coherent responses. Tobramycin generally induced a stronger and more consistent transcriptional response than gentamicin across responsive models. These data identify a robust innate immune-like transcriptional signature in human proximal tubular models following repeated aminoglycoside exposure and support primary PTEC and PTL as more informative in vitro systems for mechanistic investigation of aminoglycoside nephrotoxicity.

**Supplementary Information:**

The online version contains supplementary material available at https://doi.org/10.1007/s00204-026-04494-w.

## Introduction

Aminoglycosides are a class of antibiotics characterised by amino-modified sugars (aminocyclitols) linked via glycosidic bonds. While aminoglycosides are effective in treating various infections, including urinary tract infections and pneumonia, they often cause severe adverse effects including nephrotoxicity and ototoxicity (Thy et al. 2023). Several studies have highlighted the negative impact of aminoglycosides on renal function. Neomycin has been reported as the most nephrotoxic agent among the aminoglycosides, followed by gentamicin, tobramycin, amikacin, netilmicin and streptomycin (Luft et al. 1978; Smith et al. 1980; Kaloyanides and Pastoriza-Munoz 1980; Kahlmeter and Dahlager 1984; Williams et al. 1987). The incidence and severity of impairment are influenced by a range of factors, such as age, underlying comorbidities, genetic predisposition, the type of aminoglycoside, exposure duration, dosage and route of administration (Appel 1990; Xie et al. 2011; Serio et al. 2018; Dillard et al. 2022). Gentamicin-induced toxicity ranges from 2 to 33% (Kushner et al. 2016; Hayward et al. 2018). Critically ill patients have an increased risk of developing aminoglycoside-induced toxicity. For instance, a study by Oliveira et al. showed that 60% of critically ill patients treated with gentamicin developed acute kidney dysfunction (Oliveira et al. 2009).

Despite the high incidence of aminoglycoside-associated toxicity, the precise mechanism leading to toxicity remains yet to be fully elucidated. The primary route of cellular uptake of aminoglycosides is via endocytosis mediated by megalin (*LRP2*). Megalin is a 600 kDa multi-ligand endocytic receptor with its highest expression found in the renal proximal tubule and in the absorptive cells of the inner ear (Christensen and Birn 2002; Christensen et al. 2009; Storm et al. 2016; Hosokawa et al. 2018). Ordinarily, megalin plays an important physiological role in reabsorbing albumin, vitamin-binding proteins, vitamins, lipoproteins, and hormones (Christensen and Birn 2002). Mutations in the megalin gene (LRP2) cause growth retardation due to the loss of essential vitamins in the urine through excessive proteinurea (Dachy et al. 2015). This is the underlying cause of Donnai-Barrow syndrome, also known as faciooculoacousticorenal (FOAR) syndrome (OMIM #222448). The syndrome results from a homozygous or compound heterozygous mutation in the LRP2 gene, which is located on chromosome 2q31 and encodes the protein megalin.

The specific aim of this study was to determine whether repeated exposure to the clinically relevant aminoglycosides gentamicin and tobramycin elicits a common or model/donor-dependent transcriptional injury program in human proximal tubular cells, and to identify which in vitro renal model is most informative for mechanistic studies of aminoglycoside nephrotoxicity. To address this, we compared four human proximal tubular model systems representing different levels of physiological relevance and practicality: primary proximal tubular epithelial cells (PTEC), human iPSC-derived proximal tubular-like cells (PTL), and the immortalised cell lines RPTEC/TERT1 and HK-2. The inclusion of multiple models was due to the fact that aminoglycoside toxicity depends on proximal tubular differentiation state, uptake capacity, and intracellular trafficking, all of which may differ substantially between primary cells, stem cell-derived cells, and immortalised lines. We therefore sought not only to compare gentamicin and tobramycin, but also to assess which models best capture the molecular response associated with aminoglycoside exposure. Cells were exposed repeatedly for seven days and analysed using targeted transcriptomics. The primary endpoints were the magnitude and consistency of differentially expressed genes, the identification of dominant stress and injury-associated transcriptional programs and comparison of response patterns across drugs, models and donors. Transcriptomics was used as it enables unbiased pathway-level interrogation of early cellular responses before overt cytotoxicity is necessarily apparent and can therefore reveal mechanistic signals related to innate immune activation, inflammatory signalling, and other proximal events relevant to nephrotoxicity. This approach allowed us to define both compound-specific, model-specific and donor-specific response signatures and to evaluate the suitability of commonly used renal in vitro systems for mechanistic investigation of aminoglycoside-induced injury.

## Materials and methods

### Cell culture and differentiation

The human iPSC lines SBAD3 clone 1, SFC086 clone 1, and SFC018 clone 1 were obtained from the StemBANCC consortium (Morrison et al. 2015). iPSC were routinely cultured on Geltrex (8.3 µg/cm^2^) (Gibco, A14133-02)-coated plates in mTeSR1 medium (STEMCELL Technologies, 85850) at 36.5 °C in a humidified 5% CO_2_ incubator as previously described (Meijer et al. 2023). iPSC were passaged after reaching approximately 70% confluency. Cell culture medium was replaced daily except for the day following passaging.

The procedure to differentiate iPSC into PTL is described in detail in our protocol (Meijer et al. 2023). The human iPSC lines SBAD3, SFC086, and SFC018 were differentiated into PTL (PTL1, PTL2, and PTL3, respectively) within 14 days on Geltrex-coated 24-well plates at a seeding density of 20,000 cells/cm^2^. PTL were used for treatment starting from day 14 of the differentiation. To maintain cell stability after day 14, 1 µM GW788388 hydrate (Sigma-Aldrich, SML0116) was added to PTL-B culture medium [1:1 mixture of Dulbecco’s modified Eagle medium (DMEM) (Gibco, 11966-025) and Ham’s F-12 nutrient mix (Gibco, 21765-029) supplemented with 2 mM glutamax (Gibco, 35050-038), 5 µg/mL insulin, 5 µg/mL transferrin, 5 ng/mL sodium selenite (ITS) (Sigma-Aldrich, I1884), 10 ng/mL epidermal growth factor (EGF) (Sigma-Aldrich, E9644), and 36 ng/mL hydrocortisone (Sigma-Aldrich, H0135)].

Cryopreserved primary PTEC from three different donors, PTEC #1 (RPT101002-Male), PTEC #2 (RPT101030-Male) and PTEC #3 (RPT101029-Female), were purchased from Biopredic International (Saint-Grégoire, France). The immortalised cell lines RPTEC/TERT1 (Wieser et al. 2008) and HK-2 (Ryan et al. 1994) were obtained from Evercyte GmbH (Vienna, Austria) and ATCC (Manassas, VA, USA), respectively. Primary PTEC, RPTEC/TERT1, and HK-2 were cultured in 24-well plates in PTL-B culture medium (see previous section) supplemented with 100 U/mL penicillin, 100 µg/mL streptomycin (Pen-Strep) (Sigma-Aldrich, P4333), and 0.5% fetal bovine serum (FBS) (Gibco, 10270-106) at 36.5 °C in a humidified 5% CO_2_ incubator. Primary PTEC and RPTEC/TERT1 were cultured for a minimum of 12 days to allow for maturation (Aschauer et al. 2013). HK-2 cells were used once they reached 100% confluency.

### Aminoglycoside treatment

Gentamicin and tobramycin were purchased from Sigma-Aldrich (CAS numbers 1405-41-0 and 49842-07-1, respectively). Stock solutions at 1 g/mL were prepared in deionised water. Cell culture medium for all cell models was replaced 24 h prior to chemical exposure with medium free of FBS and Pen-Strep. Both FBS and Pen-Strep were also excluded during the exposure. All cell models were treated with gentamicin or tobramycin at concentrations of 12 µg/mL (target peak concentration (Cmax) (Hartman et al. 2020; Hollander et al. 2023), 250 µg/mL (approximately 20× Cmax), or 450 µg/mL (approximately 40× Cmax) following a repeated-dosing regimen: cells were exposed to either plain culture medium (negative control) or medium supplemented with the respective aminoglycoside concentrations (treatment), with medium renewal for all conditions every 24 h for seven consecutive days (i.e. 168 h total). All cell types were cultured and treated in three independent batches (each batch represents one replicate).

At the end of the treatment period, cells were washed once with phosphate-buffered saline (PBS) and lysed with 300 µL/well TempO-Seq lysis buffer. The lysates were stored at – 80 °C before shipment to BioClavis (Glasgow, UK) for subsequent analysis. The TempO-Seq transcriptomics assay was performed using the EU-ToxRisk v2.1 panel (3565 probes representing 3257 genes) with standard internal quality control tests as previously described (Limonciel et al. 2018; Wellens et al. 2021). The provided output file contained raw counts per probe per sample.

### Bioinformatics

Raw TempO-Seq counts per probe per sample were obtained from BioClavis following profiling with the EU-ToxRisk v2.1 panel. Differential expression analysis was performed in R using the DESeq2 package. Count data were normalized using the DESeq2 size-factor method and analysed separately for each model, compound, and concentration relative to the corresponding vehicle control. Probe-level differential expression statistics, including log2 fold change, Wald test P value, and Benjamini–Hochberg adjusted P value (padj), were extracted from the DESeq2 results tables. Unless otherwise stated, genes were considered significantly differentially expressed when they satisfied the predefined thresholds used for the corresponding downstream analysis. For visualization of representative gene expression profiles, DESeq2-normalized counts were used directly without log transformation.

For transcription factor enrichment genes significantly upregulated in at least one DESeq2 contrast (padj < 0.01, log2FC > 1) were compiled into a union gene set after collapsing probe identifiers to gene symbols. This gene set was subjected to transcription factor target enrichment analysis to determine whether differentially expressed genes were enriched among known targets of specific regulators, using pre-defined gene lists from literature for ISGF3 (STAT1/STAT2/IRF9), IRF7, IRF1, NF-κB, NFE2L2, HIF1A, and ATF4/DDIT3 (Table 1). Enrichment of each regulator set was tested using a one-sided Fisher’s exact test against the expressed-gene background in the DESeq2 table, followed by Benjamini–Hochberg multiple-testing correction.

## Results

**Fig. 1:**
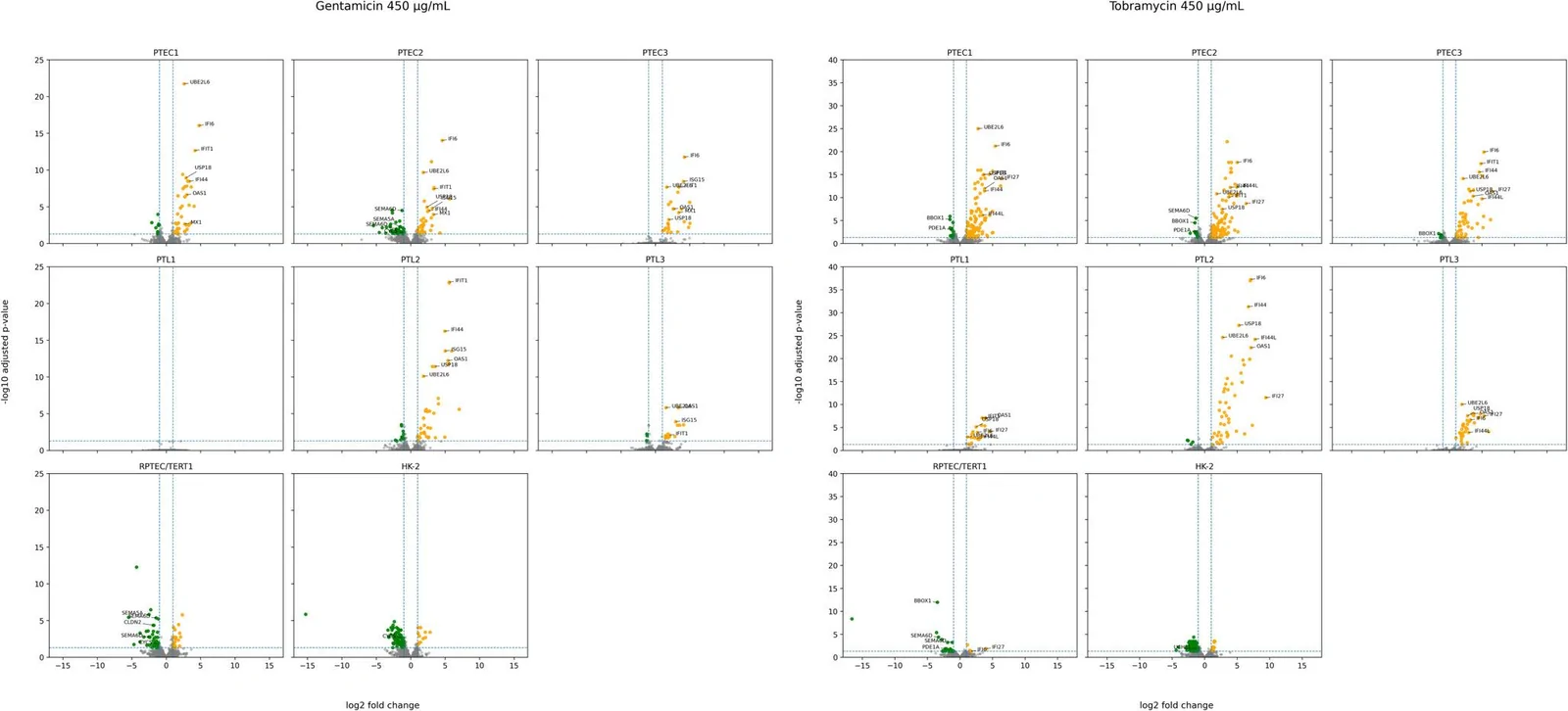
Volcano plots of aminoglycoside-induced differential gene expression in human proximal tubular models. Multi-panel volcano plots are shown for gentamicin (450 µg/mL) and tobramycin (450 µg/mL) in PTEC1–3, PTL1–3, RPTEC/TERT1, and HK-2 cells. Each point represents one gene, plotted by log2 fold change versus −log10 adjusted *P* value. Dashed vertical lines denote log2FC = ± 1, and the dashed horizontal line denotes padj = 0.05. Genes satisfying padj < 0.05 and |log2FC| ≥ 1 are highlighted, while non-significant genes are shown in grey

Repeated exposure to gentamicin and tobramycin for seven consecutive days induced marked model-dependent transcriptional responses across the proximal tubular cell systems. To visualise the overall magnitude and direction of these responses at the highest concentration tested, volcano plots were generated for all models following treatment with 450 µg/mL gentamicin or 450 µg/mL tobramycin (Fig. 1). These plots showed clear differences between the cell models in both the number and direction of significantly regulated genes. In particular, the primary PTEC models and the iPSC derived PTL models displayed prominent upregulated gene signatures, whereas RPTEC/TERT1 and especially HK-2 exhibited more divergent profiles with relatively greater downregulation. PTL from donor 1, was unresponsive to gentamicin, but was responsive to tobramycin. Recurrent upregulated genes in primary PTEC and PTL belonged to a common interferon-associated transcriptional program. Induced genes included IFI27, IFI6, IFIT1, IFIT2, IFIT3, IFITM1–3, ISG15, MX1, OAS1, UBE2L6, USP18, IFI44, IFI44L, HERC5, HERC6, RSAD2, DDX60, PLSCR1, SAMD9L, TAP1, PSMB9, and XAF1, indicating convergence on a broad interferon-stimulated gene response. In addition, genes associated with inflammatory signalling, including CCL2, CCL20, CXCL1, CASP1, BIRC3, C3, CFB, and CSF1, constituted a secondary branch of the response, which was more prominent in primary PTECs. The immortalised models did not recapitulate this response to the same extent. Instead, RPTEC/TERT1 showed a more distinct transcriptional profile, while HK-2 showed comparatively weaker pathway-level coherence and fewer enriched pathways.

To organise the transcriptional patterns observed across the volcano plots, genes that were significantly upregulated in at least one contrast were subjected to targeted regulator analysis using curated transcription factor target networks. Since the inferred targets of ISGF3 (STAT1/STAT2/IRF9), IRF7, and IRF1 showed extensive overlap in this dataset, these regulators were grouped into a single interferon-stimulated gene module, while NF-κB was retained as a distinct inflammatory module (Table 1).

**Table 1:** Major transcriptional response modules associated with aminoglycoside exposure in human proximal tubular models

Transcriptional module	Primary stimulus	Principal DNA-binding elements	Biological outcome	Induced genes
Interferon Stimulated genes (ISG, ISGF3, IRF7, IRF1)	Type I interferon signaling, viral sensing, and interferon-responsive immune signaling	ISRE, IRF-E, GAS-like elements, and type I IFN gene promoters	Induction and amplification of the interferon-stimulated gene program, antiviral defense, and antigen-presentation-associated responses	CMPK2, DDX60, EPSTI1, GBP1, HERC5, HERC6, IFI27, IFI44, IFI44L, IFI6, IFIT1, IFIT2, IFIT3, IFITM1, IFITM2, IFITM3, ISG15, ISG20, MX1, NLRC5, OAS1, PARP14, PLSCR1, PSMB9, RSAD2, SAMD9L, SP110, TAP1, TNFSF10, TRIM22, UBE2L6, USP18, WARS, XAF1
NF-κB inflammatory module	Pro-inflammatory cytokines and cellular stress	κB response elements	Inflammatory gene expression	BIRC3, C3, CASP1, CCL2, CCL20, CEBPB, CEBPD, CFB, CSF1, CXCL1, FAS, TNFSF10

**Fig. 2:**
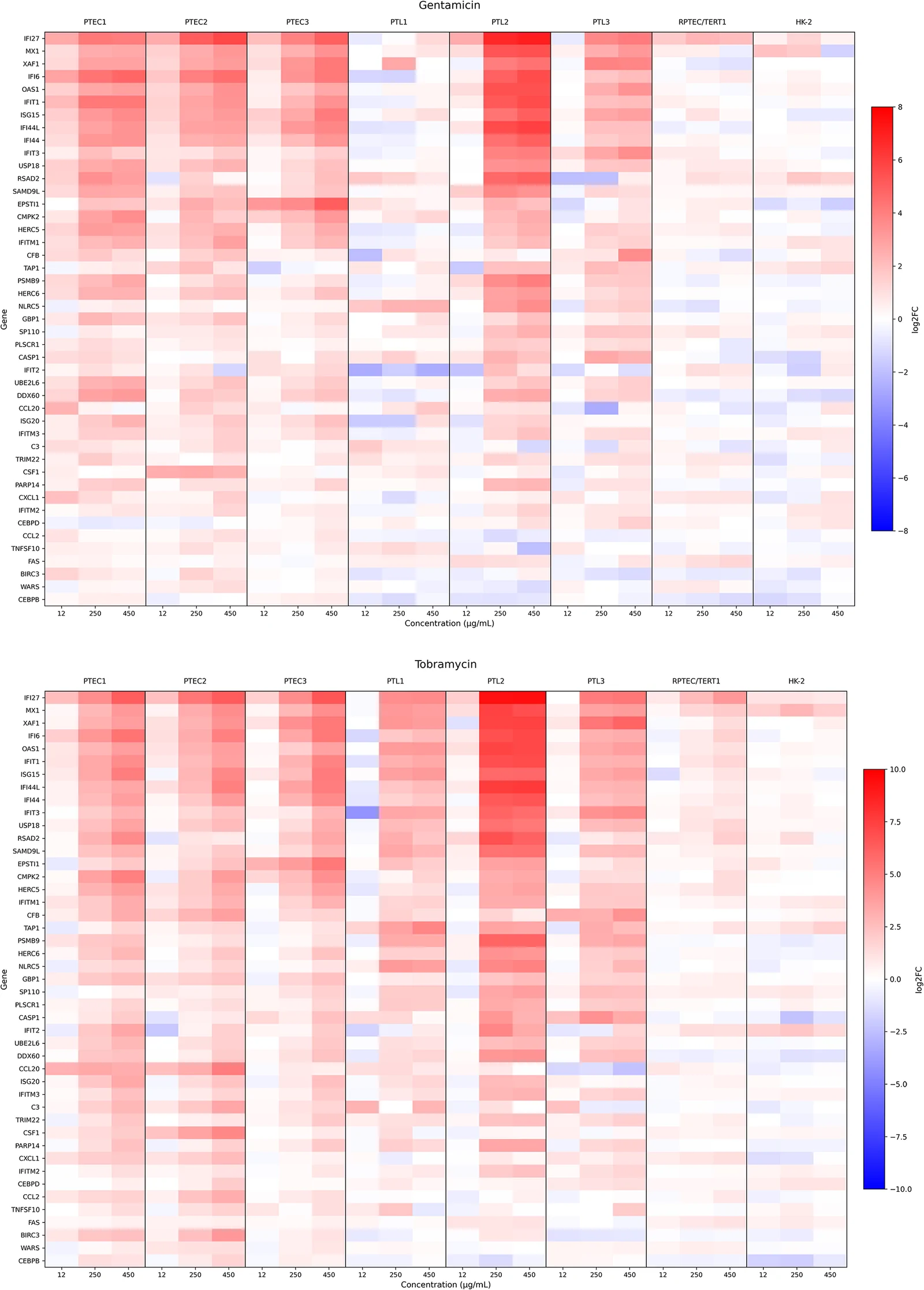
Heatmaps of TF-network-responsive genes induced by gentamicin and tobramycin. Separate heatmaps for gentamicin and tobramycin show the expression patterns of genes in the filtered union of the top four enriched transcription factor networks (STAT1/STAT2–IRF9, IRF7, IRF1, RELA/NFKB1) that were upregulated in at least one contrast (padj < 0.01, log2FC > 1). Rows correspond to genes, and columns correspond to PTEC, PTL, RPTEC/TERT1, and HK-2 at 12, 250, and 450 µg/mL. Values are plotted as the mean log2 fold change within each model class, relative to vehicle control. Red denotes higher expression, blue denotes lower expression, and white denotes minimal change

To visualise the behaviour of module-associated genes across compounds, concentrations, and model classes, heatmaps were generated using genes from the filtered union of the top four enriched transcription factor networks (Fig. 2). Gene induction was concentration-dependent in PTEC and PTL, but PTL were less responsive at 12 µg/mL. The same genes showed substantially weaker or more variable responses in RPTEC/TERT1 and HK-2.

**Fig. 3:**
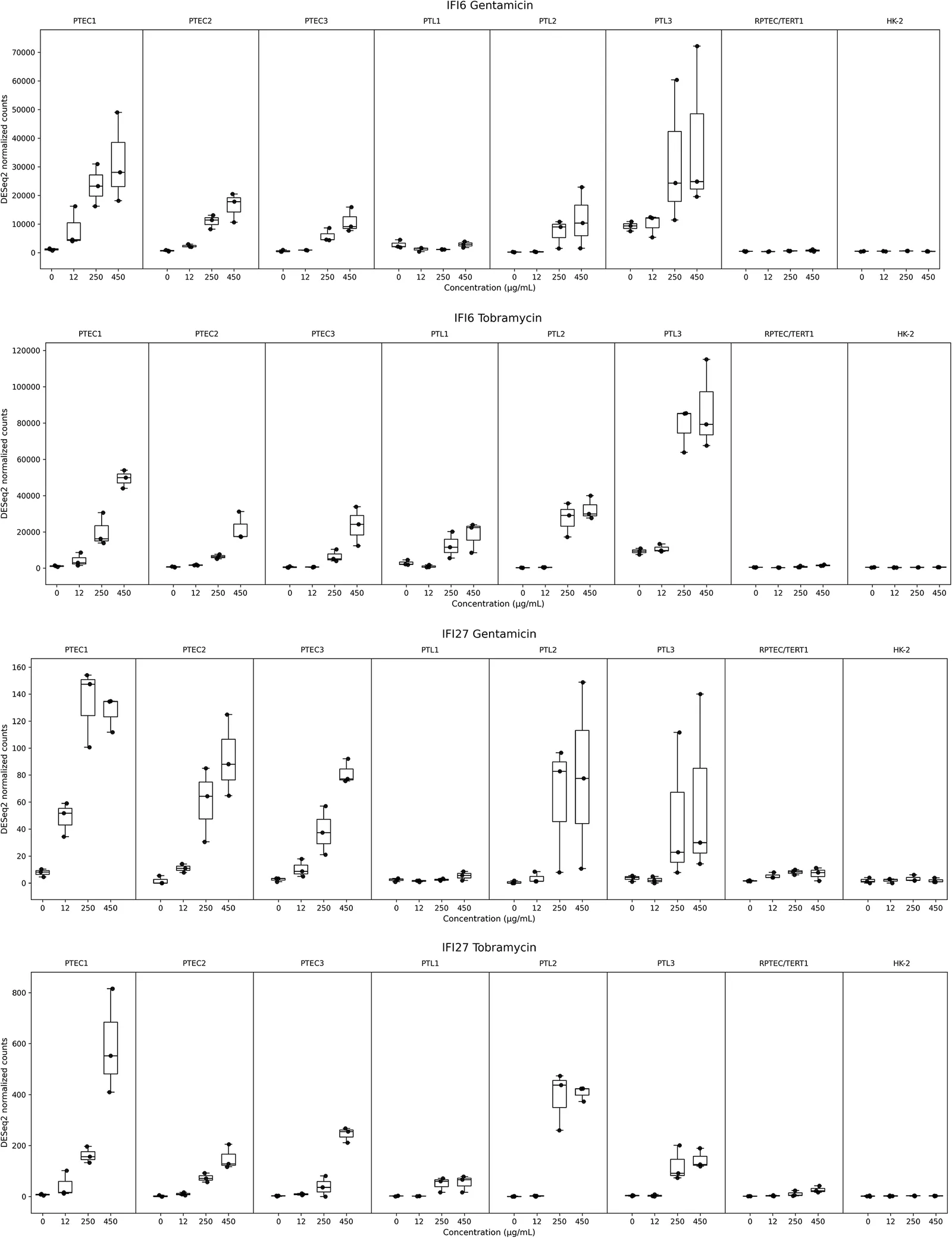

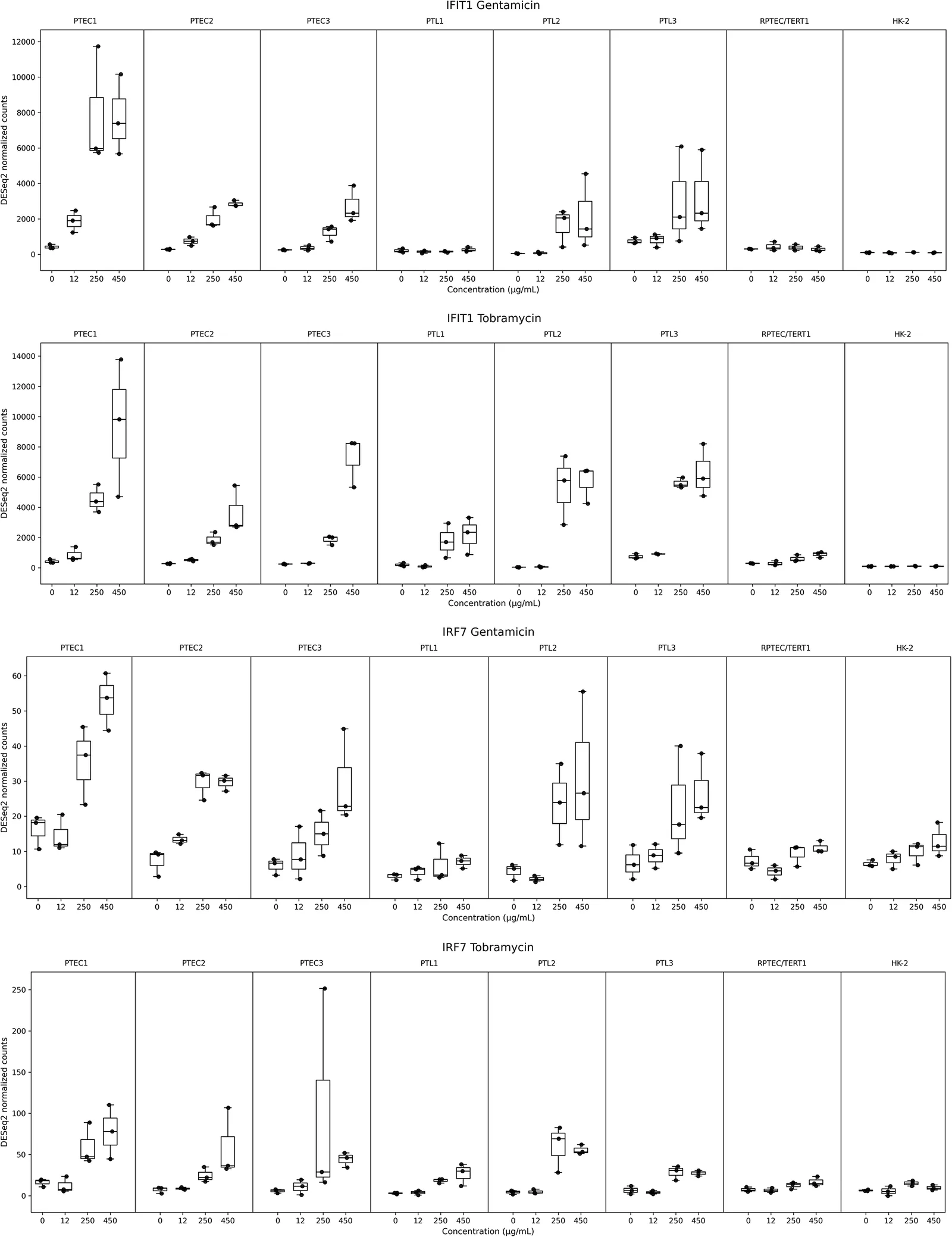

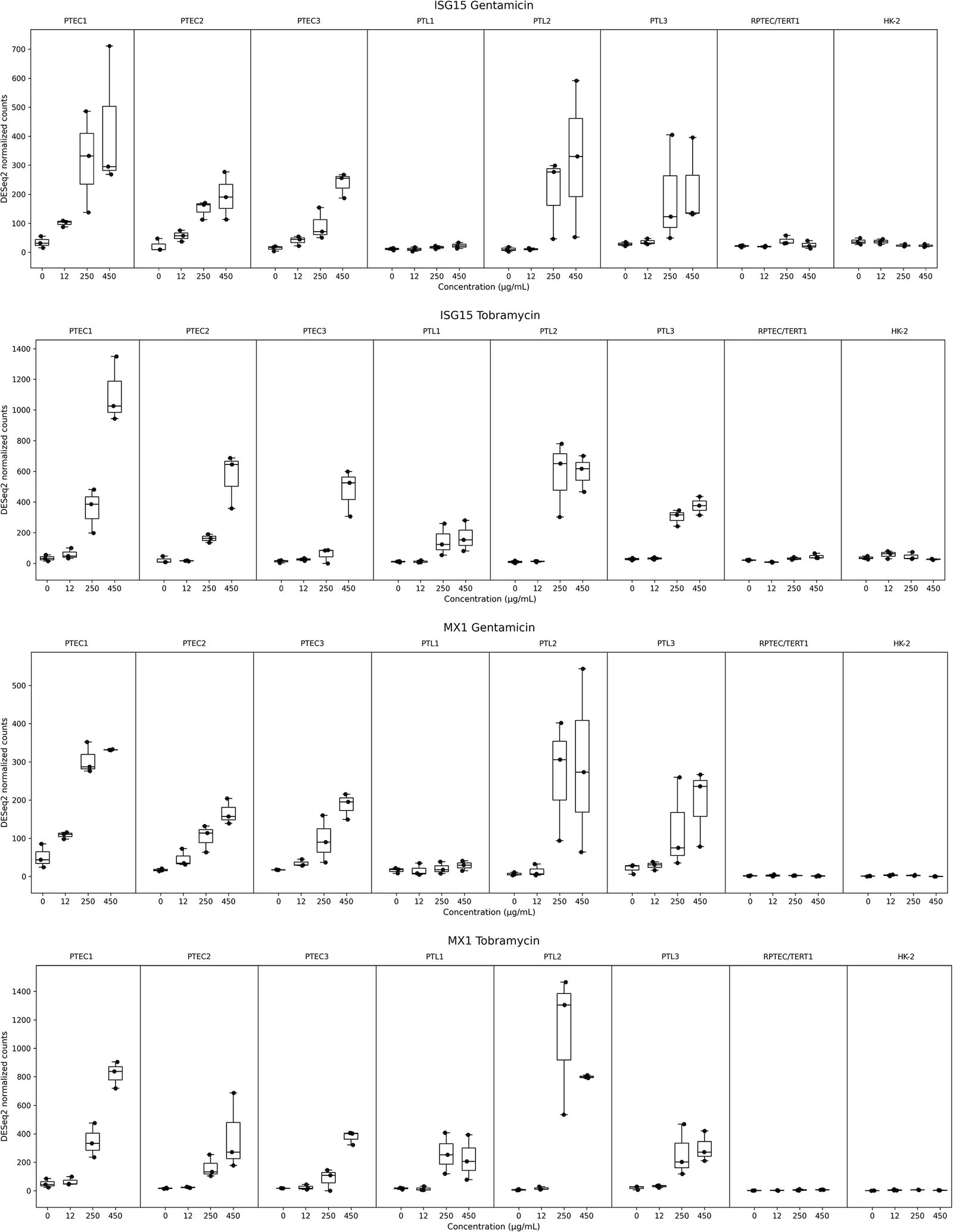

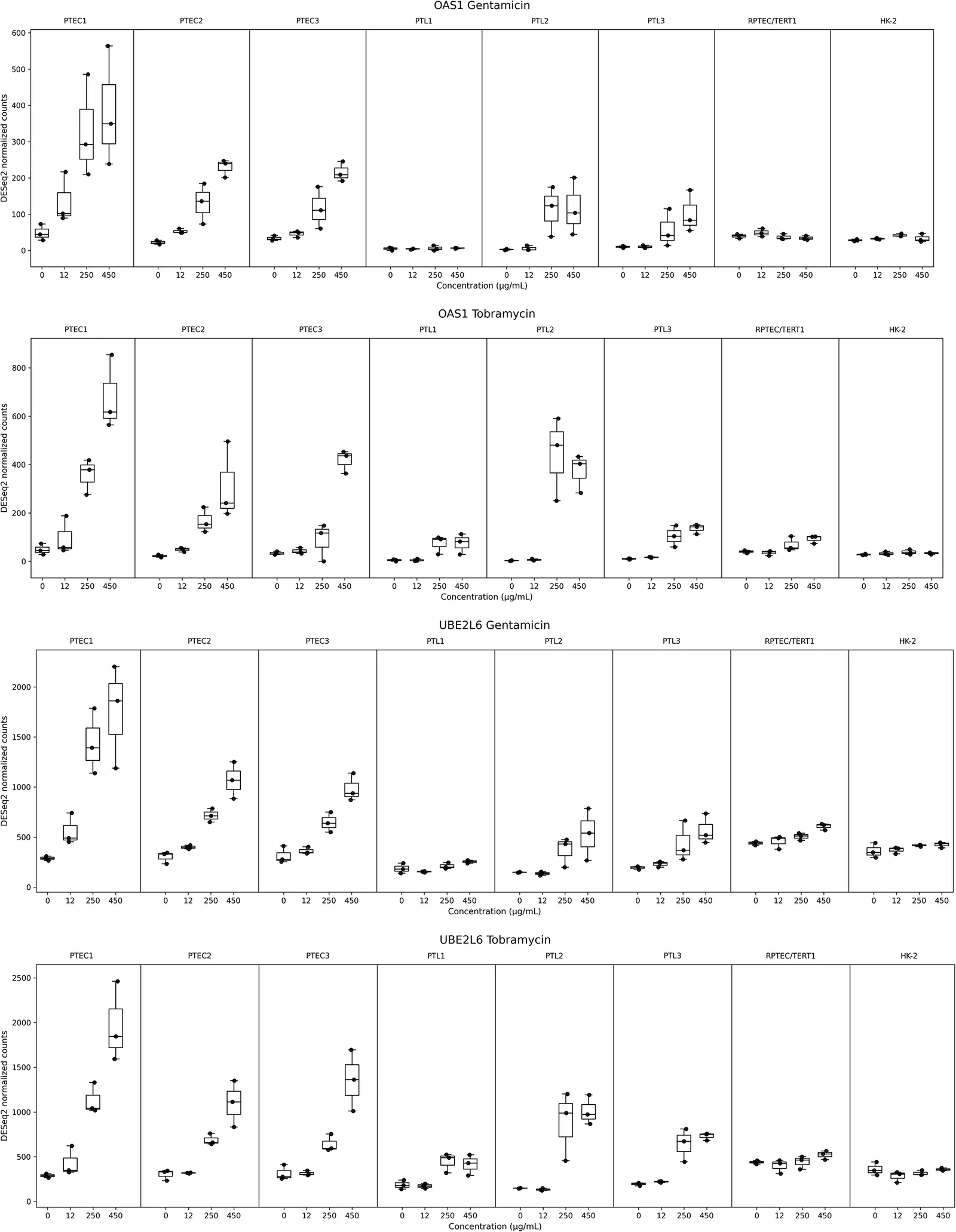
Boxplots of representative aminoglycoside-responsive genes in renal proximal tubule models. Linear expression data for selected genes in each model

To confirm that the observed transcriptional programs were also evident at the individual-sample level, boxplots were generated for representative genes using DESeq2 normalised counts (Fig. 3). Representative interferon-associated genes such as IFI27, IFIT1, ISG15, IFI6, OAS1, UBE2L6, MX1, USP18, IFI44L, HERC6, and IRF7 confirmed the strong induction of the interferon program in primary PTEC and PTL following aminoglycoside exposure.

In contrast to the robust ISG response observed in PTL models, induction of NF-κB-associated inflammatory genes was minimal, with only CASP1 and CFB showing significant upregulation in PTL2 following 250 µg/mL tobramycin exposure.

**Fig. 4:**
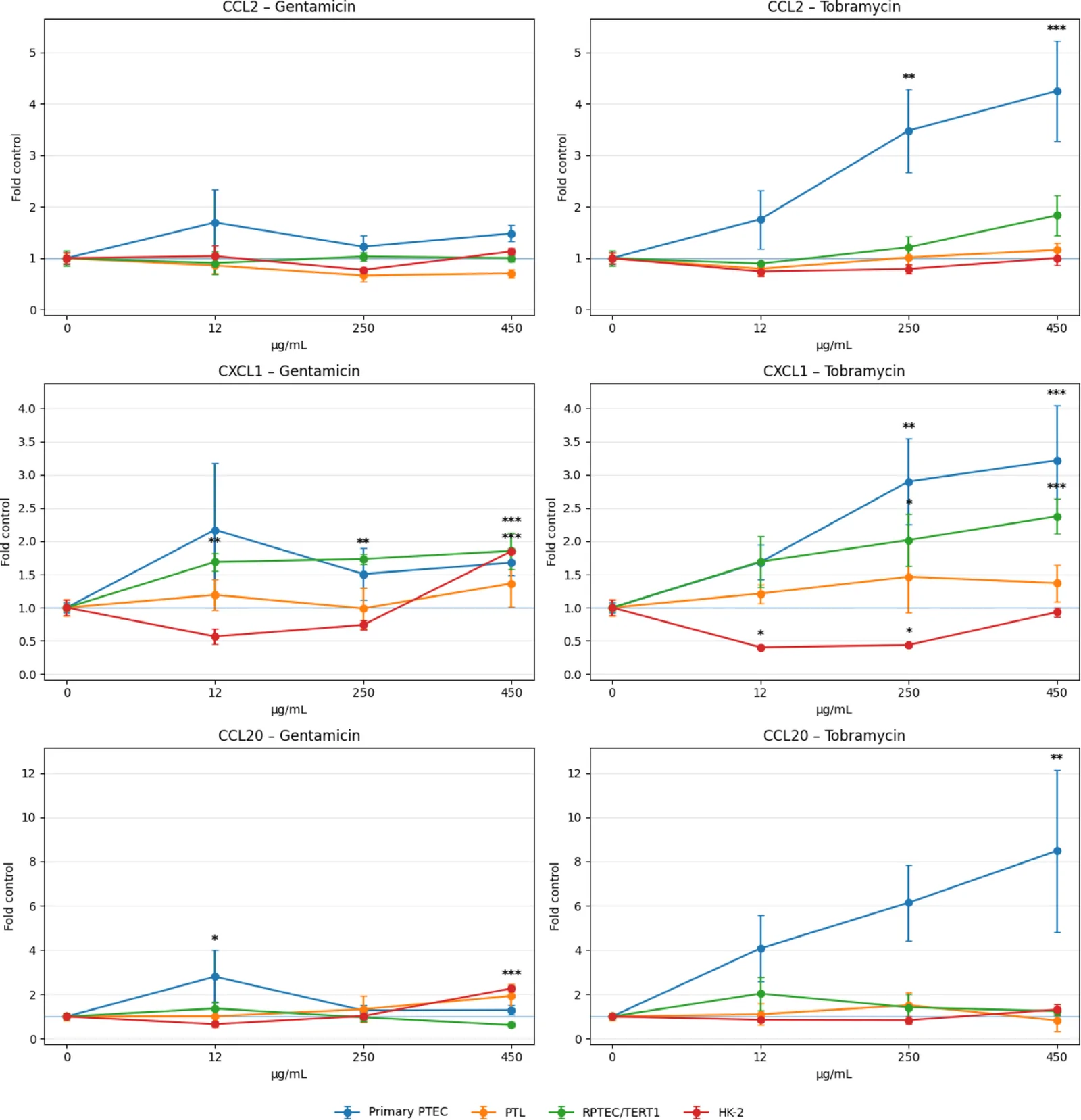
*Inflammatory related gene induction.* Concentration response plots for CCL2, CXCL1, and CCL20 in primary PTEC, PTL, RPTEC/TERT1, and HK-2 cells following repeated exposure to gentamicin or tobramycin at 12, 250, and 450 µg/mL. Expression is shown as fold of the model-matched control (0 µg/mL) using DESeq2-normalised read counts. Points represent mean ± SEM. For each gene, the gentamicin and tobramycin panels use the same y-axis scale to allow direct comparison of compound potency. Statistical significance was assessed within each model and compound by one-way ANOVA followed by Tukey’s post hoc comparison versus control; *P < 0.05, **P < 0.01, ***P < 0.001

Primary PTEC and PTL are characterised by strong induction of a shared ISG program, centered on IFI27, IFI6, IFIT1, ISG15, MX1, OAS1, UBE2L6, USP18, IFI44L, and HERC6, whereas inflammatory genes such as CCL2, CCL20 and CXCL1 form a smaller secondary branch that is much less prominent in PTL (Fig. 4).

## Discussion

In this study, we investigated the transcriptional consequences of repeated gentamicin and tobramycin exposure in a panel of human proximal tubular models comprising primary PTEC, iPSC-derived PTL, and the immortalised cell lines RPTEC/TERT1 and HK-2. Across these models, the most consistent finding was a prominent interferon-stimulated gene program. This response was most evident in the primary PTEC and PTL models, whereas RPTEC/TERT1 and HK-2 responded poorly. The strong responses observed in PTL are consistent with previous work showing that hiPSC-derived proximal tubule-like cells can yield robust and mechanistically interpretable stress-response signatures in toxicological studies (Singh et al. 2021; Snijders et al. 2021; Nunes et al. 2022; Jennings et al. 2023).

Primary PTEC and PTL showed robust upregulation of genes such as IFI27, IFI6, IFIT1, IFIT2, IFIT3, IFITM1–3, ISG15, MX1, OAS1, UBE2L6, USP18, IFI44, IFI44L, HERC5, HERC6, and RSAD2. These genes are classical interferon-stimulated genes and together define an antiviral-like transcriptional state. Targeted transcription factor target enrichment further supported this interpretation by identifying strong overlap among ISGF3 (STAT1/STAT2/IRF9), IRF7, and IRF1-associated target networks, with a smaller accompanying NF-κB-associated inflammatory branch. Since the downstream targets of ISGF3, IRF7, and IRF1 overlapped extensively in this dataset, it was decided to interpret them together as a single interferon module rather than as three separable pathways. The aminoglycoside-induced program observed here was not dominated by broad cytotoxic collapse, but by coordinated induction of genes associated with antiviral defense, interferon amplification, and antigen-presentation-related responses. The secondary inflammatory component, represented by genes such as CCL2, CCL20, CXCL1, CASP1, BIRC3, C3, and CFB, was clearly present but was substantially smaller than the interferon-associated component. The resulting picture is therefore one of an interferon-centered proximal tubular response with a smaller inflammatory branch, rather than a purely inflammatory or nonspecific injury signature.

An equally important finding is the marked divergence between model systems. Primary PTEC and PTL showed the clearest and most coherent induction of the interferon module, while RPTEC/TERT1 and HK-2 were less representative of this response. This observation is consistent with prior work indicating important functional differences between proximal tubular models, particularly with respect to endocytic uptake machinery. Aminoglycoside nephrotoxicity is closely linked to receptor-mediated uptake and intracellular accumulation, and megalin (LRP2) has long been implicated as a major route of aminoglycoside entry into proximal tubular epithelial cells (Mingeot-Leclercq and Tulkens 1999). The stronger and more coherent responses observed in primary PTEC and PTL are therefore compatible with the idea that these models retain more physiologically relevant uptake and trafficking features than RPTEC/TERT1 and HK-2. This interpretation is supported by the receptor phenotype of the immortalised models, since RPTEC/TERT1 cells were previously shown by us to lack detectable megalin expression and albumin uptake (Chandrasekaran et al. 2021), whereas HK-2 cells exhibit relatively limited megalin -associated endocytic capacity (Eshbach et al. 2017). The present transcriptomic data do not directly prove a megalin-dependent mechanism, but they are consistent with the view that biologically relevant proximal tubular phenotype is an important determinant of aminoglycoside responsiveness. The mechanism linking aminoglycoside exposure to activation of an interferon-stimulated gene program remains to be fully elucidated. However, a plausible interpretation is that aminoglycoside uptake and endosomal trapping trigger an innate immune signalling through TLRs. Previous work in murine mucosal infection models has implicated TLR3 in aminoglycoside-associated interferon responses (Gopinath et al. 2018). Representative genes from the interferon module, include IFI27, IFIT1, ISG15, IFI6, OAS1, UBE2L6, MX1, USP18, IFI44L, HERC6, and IRF7, showed clear induction in primary PTEC and PTL, while representative inflammatory genes such as CCL2, CCL20, CXCL1 and CASP1 confirmed the presence of a secondary inflammatory branch. Induction of IRF7 further supports engagement of an upstream interferon-associated regulatory circuit.

While our study captures the initial phase of cellular injury, clinical evidence also suggests that prolonged exposure to aminoglycosides shifts the pathology from primary tubular toxicity to a secondary, persistent interstitial inflammation. In clinical settings, the nephrotoxicity of gentamicin and tobramycin is fundamentally characterised by a robust, secondary inflammatory response that extends beyond initial tubular necrosis. Observations in renal biopsies and experimental models demonstrate that both compounds trigger the activation of the NF-κB signaling pathway within proximal tubular cells, resulting in the elevated secretion of pro-inflammatory cytokines such as TNF-α, IL-6, and IL-1β (Lopez-Novoa et al. 2011). The clinical nephrotoxicity of gentamicin and tobramycin is defined by a transition from acute cellular injury to a sustained inflammatory state that can progress to chronic kidney disease (CKD). Recent longitudinal studies demonstrate that even a short course of aminoglycoside treatment triggers the activation of the TLR4/NF-κB system and the NLRP3 inflammasome (Albino et al. 2021). Within this context, the higher potency observed for tobramycin in primary human PTEC and human iPSC-derived tubular models likely correlates with a more aggressive initial activation of these innate immune pathways, mediated by high-affinity binding to the megalin receptor CR10 domain (Dagil et al. 2012).

In summary, repeated exposure to gentamicin and tobramycin induced a pronounced and coherent interferon-stimulated gene response in primary human PTEC and iPSC-derived PTL models, accompanied by a smaller NF-κB-associated inflammatory component. In contrast, RPTEC/TERT1 and HK-2 showed more divergent and less representative responses. These findings identify an innate immune-like transcriptional signature as a major feature of aminoglycoside exposure in human proximal tubular models and support the use of primary PTEC and iPSC-derived PTL as more informative in vitro systems for mechanistic studies of aminoglycoside nephrotoxicity.

## Supplementary Information

Below is the link to the electronic supplementary material.

Supplementary Material 1

## Data Availability

No datasets were generated or analysed during the current study. Processed transcriptomic data supporting the findings of this study are provided in the manuscript and its Supplementary Materials. Any additional information is available from the corresponding author upon reasonable request.
